# CFTR is involved in the regulation of glucagon secretion in human and rodent alpha cells

**DOI:** 10.1038/s41598-017-00098-8

**Published:** 2017-03-07

**Authors:** Anna Edlund, Morten Gram Pedersen, Andreas Lindqvist, Nils Wierup, Malin Flodström-Tullberg, Lena Eliasson

**Affiliations:** 1 Unit of Islet Cell Exocytosis, Lund University Diabetes Centre, Department of Clinical Sciences in Malmö, Lund University, CRC, SUS Malmö, Malmö, Sweden; 2 Department of Information Engineering, University of Padova, Via Gradenigo 6/B, 35131 Padova, Italy; 3 Unit of Neuroendocrine Cell Biology, Lund University Diabetes Centre, Department of Clinical Sciences in Malmö, Lund University, CRC, SUS Malmö, Malmö, Sweden; 4 Center for Infectious Medicine, Department of Medicine, Karolinska Institutet, Huddinge University Hospital, Stockholm, Sweden

## Abstract

Glucagon is the main counterregulatory hormone in the body. Still, the mechanism involved in the regulation of glucagon secretion from pancreatic alpha cells remains elusive. Dysregulated glucagon secretion is common in patients with Cystic Fibrosis (CF) that develop CF related diabetes (CFRD). CF is caused by a mutation in the Cl^−^ channel Cystic fibrosis transmembrane conductance regulator (CFTR), but whether CFTR is present in human alpha cells and regulate glucagon secretion has not been investigated in detail. Here, both human and mouse alpha cells showed CFTR protein expression, whereas CFTR was absent in somatostatin secreting delta cells. CFTR-current activity induced by cAMP was measured in single alpha cells. Glucagon secretion at different glucose levels and in the presence of forskolin was increased by CFTR-inhibition in human islets, whereas depolarization-induced glucagon secretion was unaffected. CFTR is suggested to mainly regulate the membrane potential through an intrinsic alpha cell effect, as supported by a mathematical model of alpha cell electrophysiology. In conclusion, CFTR channels are present in alpha cells and act as important negative regulators of cAMP-enhanced glucagon secretion through effects on alpha cell membrane potential. Our data support that loss-of-function mutations in *CFTR* contributes to dysregulated glucagon secretion in CFRD.

## Introduction

Glucagon is the main hyperglycemic hormone in the body and is released during fasting and extensive exercise. The hormone is released from pancreatic alpha cells, situated in the islet of Langerhans together with insulin secreting beta cells and somatostatin secreting delta cells. The islets of Langerhans are clusters of cells which are spread throughout the exocrine part of pancreas and constitute the endocrine part of the organ. Currently, we have not reached the full understanding of the cell physiology regulating glucagon secretion, and both intrinsic and paracrine regulation has been suggested to be involved^1, 2^. For example, it has been hypothesized that glucagon is released as a result of an intermediate whole-cell K_ATP_-conductance, i.e. only part of the K_ATP_-channels are open, at low glucose concentration resulting in activation of voltage-dependent Na^+^ and Ca^2+^ channels^3, 4^. The resulting influx of Ca^2+^ initiates exocytosis of glucagon-containing granules. According to this hypothesis glucagon secretion is maximally inhibited at a glucose concentration of ~5–6 mM as a consequence of closure of the K_ATP_-channel and inactivation of voltage-dependent Na^+^ channels^5^. However, the regulation of alpha cell electrical activity and secretion has also been suggested to involve store-operated channels^6^. A recent mathematical model of electrical activity in alpha cells suggests that glucagon secretion is most likely controlled by a combination of the two mechanisms^7^. SGLT2 Na^+^-glucose co-transporters have also been suggested to be involved in stimulus-secretion coupling in alpha-cells^8, 9^. Paracrine inhibition of glucagon secretion involves zinc^10^ and GABA^11^ released by beta cells, and somatostatin released from delta cells^12, 13^.

Somatostatin is known to inhibit both insulin and glucagon secretion^14, 15^. Pancreatic delta cells secrete somatostatin in response to increased glucose levels, and this has been suggested to involve the activation of calcium induced calcium release (CICR)^16^. Paracrine effects on somatostatin secretion involve stimulation by glucagon and insulin when alpha- and beta cells are active^17–20^.

The cystic fibrosis transmembrane conductance regulator (CFTR) is a Cl^−^ channel that belongs to the family of ABC-transporter proteins and is activated by cAMP^21^. In accordance with the function of many ABC-transporters, CFTR, aside from conducting Cl^−^ ions through its channel pore, can also act as a regulator of other ion-channels and proteins^22^. Mutations in the gene encoding the CFTR channel impair the ion channel function and causes cystic fibrosis (CF), a disease that is characterized by malfunction in secretion by the epithelium in a variety of organs, including the respiratory tract, exocrine pancreas, sweat glands and the intestine^23^. Today patients with CF live longer and many develop Cystic Fibrosis Related Diabetes (CFRD), which is associated with impaired insulin secretion^24, 25^. The reduced insulin secretion has been suggested to at least in part be due to destruction of the beta cells by the damaged exocrine cells^24, 26^. However, recent studies in patients and animal models have suggested a direct role of CFTR in the control of insulin secretion^24, 27–30^, and we and others have recently shown presence of CFTR in pancreatic beta cells and its direct involvement in the regulation of processes controlling insulin secretion^31, 32^.

Here, we have investigated if CFTR is present in alpha- and delta cells and involved in the intrinsic mechanisms regulating hormone secretion in human and mouse alpha- and delta-cells. For this purpose, we have used patch-clamp registrations of current activation, hormone secretion assays, capacitance measurements as a measure of exocytosis, and mathematical modelling to interpret our findings.

## Results

### CFTR protein is expressed in human and mouse alpha cells

**Figure 1:**
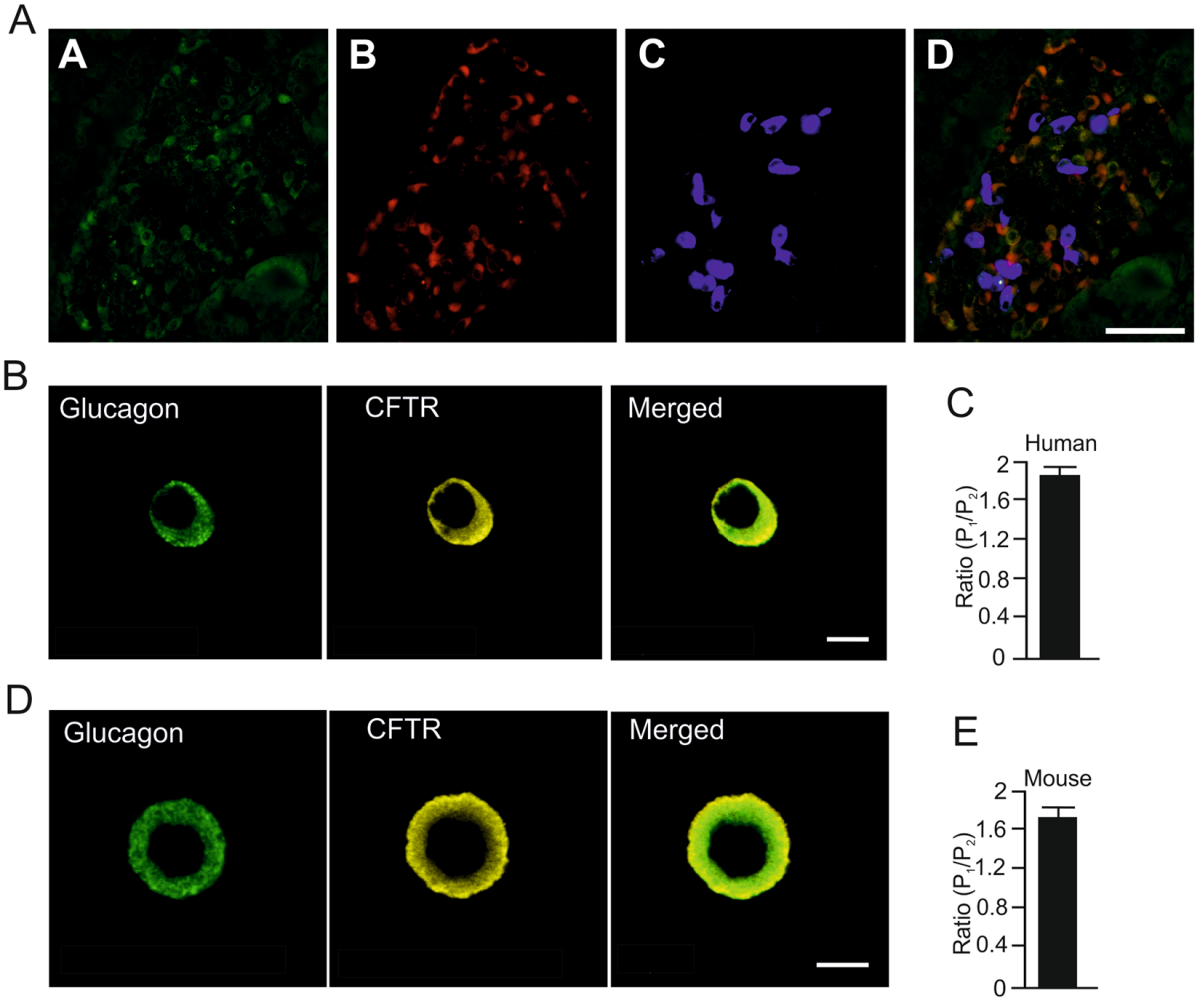
CFTR is present in mouse and human alpha cells, but absent in delta cells. (**A**) Immunofluorescence of CFTR in sections of human pancreas. Immunostaining for CFTR (**A** **.A**), glucagon (**A** **.B**) and somatostatin (**A** **.C**), the overlay showing co-localization (**A** **.D**). Representative images from 3 donors and 20 islets from each donor that were investigated. (**B**) Confocal immunolocalization of CFTR (yellow) and glucagon (green) in fixed single pancreatic human alpha cell. (**C**) Mean intensity ratio (P_1_/P_2_) of CFTR antibody in the plasma membrane (P_1_) compartment compared to cytosolic compartment (P_2_) in human alpha cells (n = 30 cells from N = 3 donors). (**D**) As in B, but image showing a single mouse alpha cells. (**E**) As in C, but the estimate was made in mouse alpha cells (n = 7 cells from N = 2 mice). Plasma membrane region was 0.5 μm. White scale bar indicates 50 μm in A and 5 μm in B and D.

First, we used human pancreas sections to investigate the presence of CFTR in human islet alpha-and delta-cells using immunohistochemistry. We found that CFTR is expressed in human alpha cells but not in delta cells (Fig. 1A). We next moved on to investigate the CFTR localization in human alpha cells on the single cell level. The cells were stained for glucagon and CFTR and investigated using a confocal microscope (Fig. 1B). This enabled determination of subcellular localization of CFTR immunostaining, e.g. in the plasma membrane region compared to the cytosolic region (P_1_/P_2_). The CFTR signal was almost two-fold higher in the plasma membrane region compared to the cytosol in human alpha cells (Fig. 1C). Similar results were obtained in mouse alpha cells (Fig. 1D–E).

### CFTR-inhibitors enhance glucose-regulated glucagon secretion

**Figure 2:**
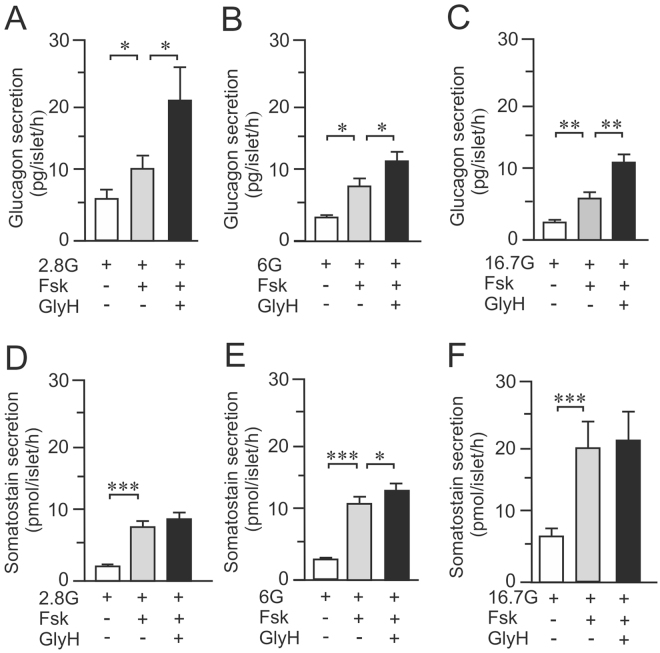
Effects on glucagon- and somatostatin secretion after CFTR inhibition in human islets. (**A**) Glucagon secretion measured in human islets (N = 9) incubated for 1 h at 2.8 mM glucose (2.8G) in the presence or absence of 10 μM forskolin (Fsk) and 50 μM GlyH-101 (GlyH). (**B**) Same as in A, but measurements were performed at 6 mM glucose (6G) (N = 9). (**C**) Same as in A, but measurements were performed at 16.7 mM glucose (16.7G) (N = 9). (**D–F**) Same as A–C, but somatostatin secretion was measured (N = 11). *p < 0.05 **p < 0.01, ***p < 0.001.

We were interested in determining if CFTR is involved in the regulation of glucagon secretion in human islets. Isolated islets from human cadaver donors were incubated at 2.8 mM glucose (Fig. 2A). Forskolin, known to increase intracellular cAMP, was used to activate CFTR, as well as other cAMP-dependent pathways in the alpha cell^6, 33^. Forskolin induced a 2-fold increase in glucagon secretion that was further increased in the presence of the CFTR inhibitor, GlyH-101. Similar results were observed when glucagon secretion was measured from islets incubated at 6 mM (Fig. 2B) and 16.7 mM (Fig. 2C) glucose. To address whether there are off target effects of GlyH-101 on alpha cells, mouse and human islets were treated with siRNA against CFTR and glucagon secretion was measured (Suppl. Fig. 1). These data confirmed that the effect of GlyH-101 on glucagon secretion is indeed via CFTR.

We also measured release of somatostatin from the same islets. At all glucose concentrations (Fig. 2D–F) forskolin stimulated an increase in somatostatin secretion that was unaffected by inhibition of CFTR (Fig. 2D–F).

**Figure 3:**
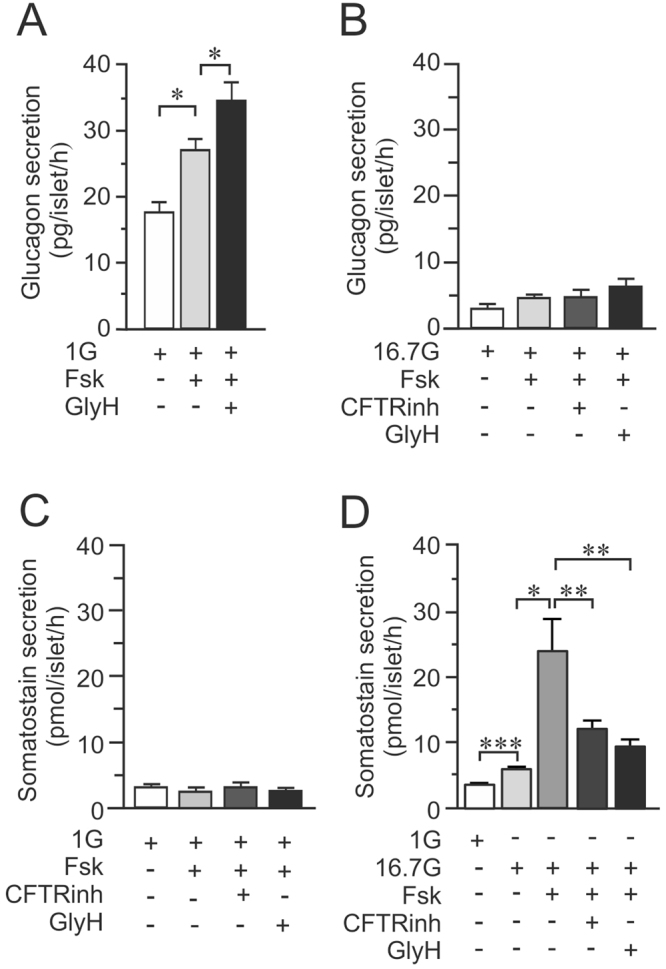
CFTR inhibition on glucagon and somatostatin secretion in mouse islets. (**A**) Glucagon secretion from mouse islets incubated for 1 h in 1 mM glucose (1G), 10 μM forskolin (Fsk) and/or 50 μM GlyH-101 (GlyH) as indicated (N = 4). (**B**) As in A, but experiments where performed at 16.7 mM glucose (16.7G) and also in presence of 40 μM CFTRinh-172 (CFTRinh) as indicated (**C**) As in A but somatostatin secretion was measured (N = 4) (**D**) As in C, but experiments where performed in presence of 16.7 mM glucose (N = 9). *p < 0.05, **p < 0.01.

In mouse islets, glucagon secretion at 1 mM glucose was enhanced in presence of forskolin, this was further increased by ~30% in presence of GlyH-101 (Fig. 3A), in accordance with the results in human islets. However, at 16.7 mM glucose glucagon secretion remained virtually unchanged with or without CFTR-inhibition (Fig. 3B). Glucose-stimulated somatostatin secretion in mouse islets at 16.7 mM was significantly increased by forskolin and both GlyH-101 and CFTRinh-172 significantly reduced the amplifying effect of forskolin on glucose-stimulated somatostatin secretion (Fig. 3D).

### CFTR current detected in human and mouse single alpha cells

**Figure 4:**
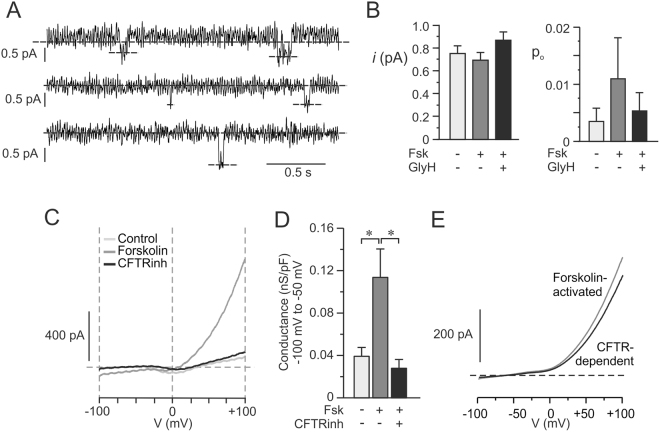
Effects of CFTR inhibition on currents single human and mouse alpha cells. (**A**) Representative recordings showing the single-channel activity of CFTR in single human alpha cells using the cell-attached mode. Traces shown are in presence of 10 μM forskolin (Fsk) and the voltage was clamped at −50 mV. Dotted lines (over the whole traces) indicate the closed channel state and downward deflections correspond to channel openings. **(B)** Single-channel current amplitude (*i;* left) and open probability (p_o_; right) in the absence (Ctrl, light grey) or presence of 10 μM Fsk (grey) and in the simultaneous presence of Fsk and 50 μM Gly-H-101 (GlyH, black). (n = 6 cells from N = 2 donors). **(C)** Representative recordings showing whole-cell currents in single mouse alpha cells upon stimulation with voltage ramps from −100 mV to +100 mV in a single alpha cell performed in the absence (Ctrl, light grey) or presence of 10 μM forskolin (Fsk, grey) and in the simultaneous presence of forskolin and CFTRinh-172 (CFTRinh, black). Current ramps were applied before and every fourth minute after the application of forskolin until steady state was achieved. **(D)** Bar graph of the membrane conductance at negative voltages from −100 mV to −50 mV in alpha cell under the different conditions as in C (n = 5). **(E)** Calculated forskolin-activated and CFTR-dependent current from data in C. Currents shown are mean of n = 5 cells from 4 mice. *p < 0.05.

To determine if human alpha cells have active CFTR currents we performed patch-clamp cell-attached single channel recordings. The voltage was held at −50 mV and continuous current activity was measured. It is not easy to detect CFTR single channel current in primary cells, but we managed to detect current opening of the size (~0.7 pA) and direction typical of CFTR^34^, suggesting the presence of CFTR in alpha cells (4A-B). The open probability (p_o_) of the current increased with forskolin (n = 6 cells from 2 donors), and in three of the cells investigated GlyH-101 was added and found to decrease p_o_ to the level of control. We further performed whole cell current measurements in single mouse alpha cells. In these experiments a whole-cell current was initiated by the application of a voltage ramp from −100 mV to +100 mV (Fig. 4C–E). In the absence of forskolin the ramp protocol evoked a minimal current, while addition of forskolin evoked a significant increase in current. The cAMP-dependent increase in current was almost totally inhibited by the CFTRinh-172 (Fig. 4C,D). The current inhibited by CFTR-inhibitors (CFTR-dependent) constituted 76 ± 9% (n = 5) of the forskolin-activated current at negative potentials (Fig. 4E). The CFTR-dependent conductance in mouse alpha cells was estimated to 61 ± 20 pS/pF at negative potentials (−100 mV to −50 mV) and the Cl^−^ reversal potential was measured to E_Cl_ −61 ± 2 mV.

### Depolarization-induced glucagon- and somatostatin-secretion is reduced by CFTR-inhibition in mouse but not in human islets

**Figure 5:**
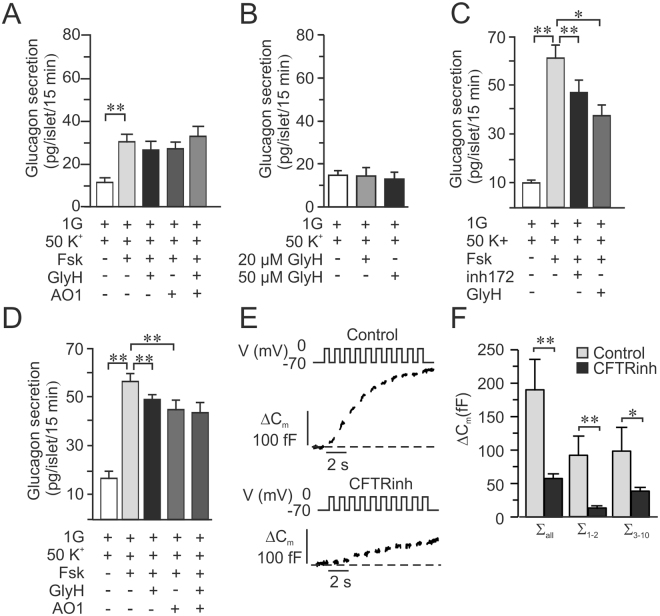
Effect of CFTR inhibition on depolarization evoked glucagon secretion in human and mouse alpha-cells. (**A**) Glucagon secretion in human islets stimulated for 15 minutes by 50 mM K^+^ in the absence and presence of 10 μM forskolin (Fsk), 50 μM GlyH-101 (GlyH) and 50 μM TMinh-AO1 (AO1) as indicated (N = 4). (**B**) Glucagon secretion in human islets stimulated for 15 minutes by 50 mM K^+^ in the absence of forskolin and in the presence of 20 μM or 50 μM GlyH-101 (GlyH) as indicated (N = 3). (**C**) Glucagon secretion in mouse islets measured after 15 min incubation at 1 mM glucose (1G) in the presence of 50 mM KCl (K^+^) and 10 μM forskolin (Fsk) and 40 μM CFTRinh-172 (CFTRinh) or 50 μM GlyH-101 (GlyH) as indicated (N = 4). (**D**) As in C, but the effect of the ANO1 inhibitor T16Ainh-AO1 (AO1) on Fsk enhanced and GlyH-inhibited glucagon secretion was investigated in mouse islets. (N = 4). (**E**) Representative traces of increases in membrane capacitance (ΔC_m_; bottom) in single mouse alpha cells when exocytosis was elicited by a train of ten 500 ms depolarizations from −70 mV to 0 mV (top). CFTR was activated by presence of 0.1 mM cAMP in the pipette solution under control conditions (Control; top) and in cells pre-incubated for 10 min with CFTRinh-172 (CFTRinh; bottom) (**F**) Summary of the increase in membrane capacitance under control conditions (n = 5) and after 10 min preincubation with CFTRinh-172 (n = 7). Data is presented as the increase in membrane capacitance evoked by all 10 pulses of the train (∑_all_), the two first pulses (∑_1–2_) or the latter eight pulses (∑_3–10_). *p < 0.05, **p < 0.01.

We have previously suggested that CFTR act together with the Ca^2+^-activated chloride channel Anoctamin 1 (ANO1) in the regulation of insulin secretion in pancreatic beta cells (Edlund *et al*.^31^), suggesting a role in exocytosis. We therefore investigated if inhibitors of CFTR (GlyH-101) and ANO1 (T16Ainh-AO1) have any effect on depolarization-induced glucagon secretion using a high concentration of K^+^. Human islets were incubated for 15 minutes in presence of 1 mM glucose and 50 mM KCl (50 K^+^). Forskolin significantly enhanced secretion 3-fold, an increase that was unaffected by the CFTR-inhibitor and the inhibitor of ANO1 (Fig. 5A). It was ascertained that GlyH-101 did not have any effect in the absence of forskolin (Fig. 5B). In rodent alpha cells the inhibitors of CFTR, GlyH-101 and CFTRinh-172, surprisingly reduced depolarization-induced secretion in presence of forskolin (Fig. 5C). Moreover, addition of the ANO1-inhibitor into the incubation media reduced K^+^ -stimulated glucagon secretion to the same extent as GlyH-101 and there was no additive effect in the simultaneous presence of GlyH-101 and T16Ainh-AO1 (Fig. 5D), suggesting that CFTR and ANO1 at least partly regulate mouse alpha cells through effects on exocytosis as has been described in beta cells. This made us measure exocytosis in single mouse alpha cells as an increase in membrane capacitance (ΔC_m_). Experiments were performed using the standard whole-cell configuration of the patch-clamp technique and the intracellular solution was supplemented with cAMP. Alpha cells were pre-incubated in the absence (control) or presence of 10 μM CFTRinh-172 for ten minutes prior to capacitance measurements and exocytosis was evoked by a train of ten 500-ms depolarisations from −70 mV to 0 mV (Fig. 5E). In summary, the total exocytotic response evoked by the train (∑_all_, Fig. 5F) was significantly reduced in cells pre-incubated with CFTRinh-172 compared to control alpha cells.

**Figure 6:**
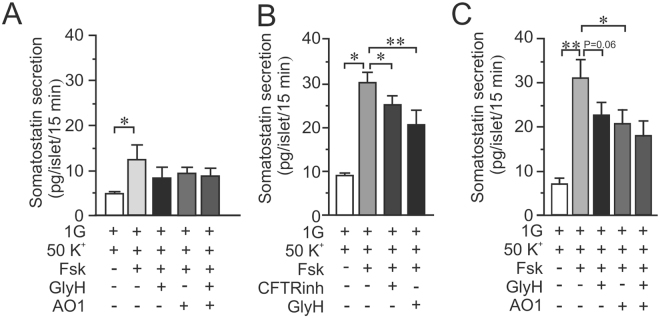
Effect of CFTR inhibition on depolarization evoked somatostatin secretion in human and mouse islets. (**A**) Somatostatin secretion in human islets measured after 15 min incubation at 1 mM glucose (1G) in the presence of 50 mM KCl (K^+^) and 10 μM forskolin (Fsk) and 50 μM GlyH-101 (GlyH) as indicated (N = 4). (**B**) Somatostatin secretion in mouse islets measured after 15 min incubation at 1 mM glucose (1G) in the presence of 50 mM KCl (K^+^) and 10 μM forskolin (Fsk) and 40 μM CFTRinh-172 (CFTRinh) or 50 μM GlyH-101 (GlyH) as indicated (N = 4). (**C**) As in B, but the effect of the ANO1 inhibitor TMinh-AO1 (AO1) on FSK enhanced and GlyH-inhibited glucagon secretion was investigated in mouse islets. (N = 4). *p < 0.05, **p < 0.01.

We measured somatostatin secretion from the same islets as used for glucagon secretion measurements in Fig. 5A,C,D to observe how depolarization-induced somatostatin secretion is affected by CFTR and ANO1 inhibition. Depolarization- induced somatostatin secretion was enhanced by forskolin in both human and mice islets (Fig. 6A–C). In human islets (Fig. 6A), addition of any of the inhibitors of CFTR or ANO1 did not affect the response to forskolin significantly. However, in mouse islets (Fig. 6B,C), inhibition of either CFTR or ANO1 reduced depolarization-induced somatostatin secretion. Moreover, there was no additive effect between the CFTR-inhibition and inhibition of the Ca^2+^ activated chloride channel (Fig. 6C).

### Simulation of alpha cell electrical activity explains the role of CFTR in alpha cells

**Figure 7:**
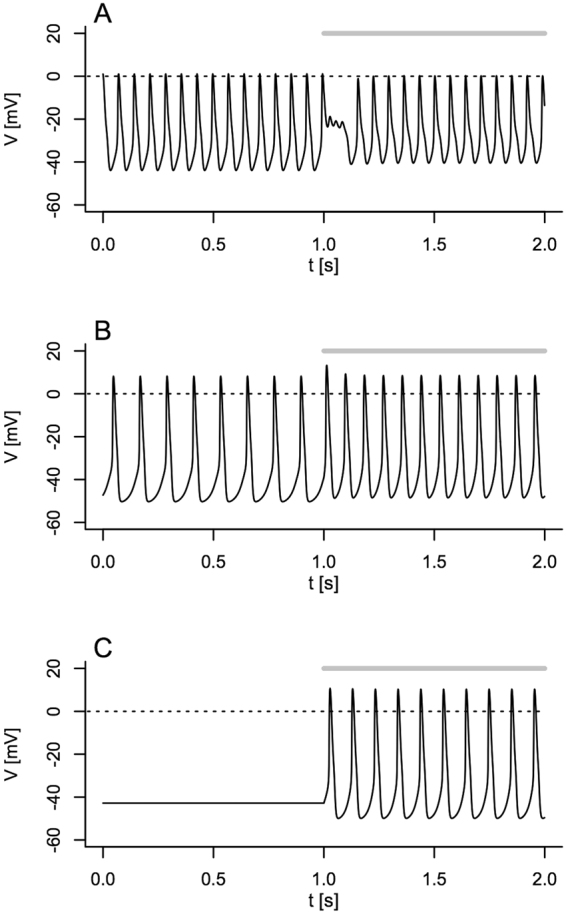
Simulation of electrical activity in alpha cells after introduction of CFTR. (**A**) Electrical activity was simulated using the model of Watts and Sherman^7^ with gKATP = 0.200 nS (=6 mM glucose^3^), gL = 0.200 nS and with gCFTR = 0.100 nS. Gray bar indicate CFTR inhibition. (**B**) As in A but gKATP = 0.270 nS (=1 mM glucose^3^) and gL = 0.150 nS^35^. (**C**) As in A but with gKATP = 0.300 nS (within experimental range at 1 mM glucose according to ref. 3).

The secretion experiments show that active CFTR channels inhibit glucagon secretion in the presence of forskolin and different concentrations of glucose. CFTR has no effects downstream of cell membrane depolarization in human alpha cells, and affect exocytosis positively in mouse alpha cells. This might contribute to the well-established cAMP-mediated effects downstream of Ca^2+^ influx^33, 35^. Together, these findings indicate that the main overall negative role of CFTR seen in the glucagon secretion experiments is due to effects on alpha cell electrical activity. To get theoretical insight into the feasibility of this hypothesis, we inserted a CFTR-dependent current based on our electrophysiological characterization into the recent alpha cell model by Watts and Sherman^7^. Our simulations showed that the effect of CFTR inhibition depends on the K_ATP_ conductance of the model cell (Fig. 7A–C). At low K_ATP_ conductance, *g*_KATP_ = 0.200 nS, corresponding to 6 mM glucose^3^, CFTR inhibition leads to a slight increase in action potential firing frequency (Fig. 7A). With more K_ATP_ channels open (*g*_KATP_ = 0.270 nS), corresponding to 1 mM glucose^3^ the result is similar, though the effect on the firing frequency is more pronounced (Fig. 7B). At slightly higher K_ATP_ conductance, *g*_KATP_ = 0.300 nS, which is still within experimental variation observed at 1 mM glucose^3^, the cell is silent with CFTR operating, but activates once CFTR is inhibited (Fig. 7C). Thus, these simulations suggest that CFTR negatively regulates alpha cell activity in two ways: (i) by reducing action potential firing, and (ii) by silencing some alpha cells that are active in the absence of the CFTR current.

## Discussion

We have in this study specifically investigated CFTR in human and mouse pancreatic alpha cells. Our data demonstrate the presence of CFTR in pancreatic alpha cells and that CFTR is involved in the regulation of cAMP-dependent glucagon secretion.

**Figure 8:**
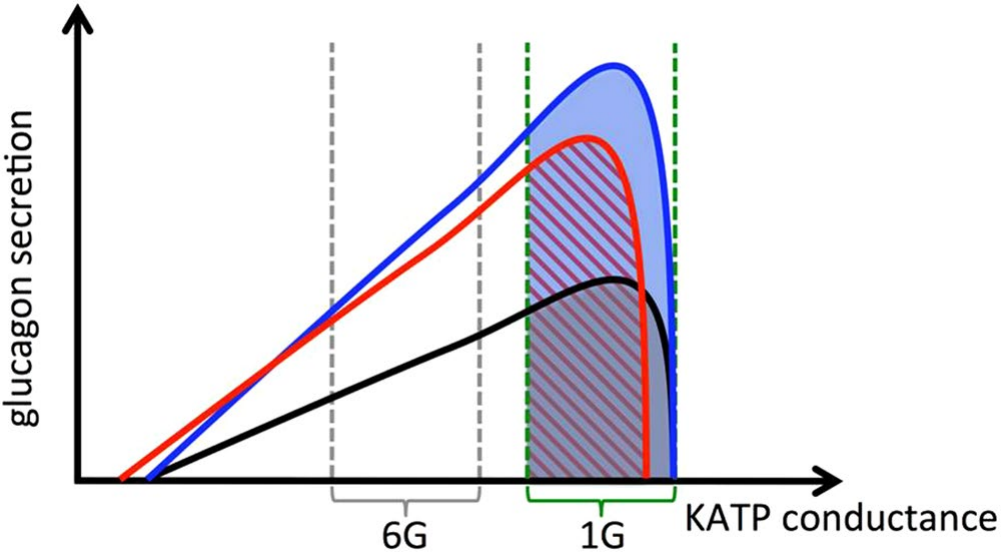
Model suggesting how CFTR affects glucagon secretion in pancreatic alpha cells. Glucose stimulated glucagon secretion results in the black curve, while the summed effect of cAMP on amplification pathways and activation of CFTR results in the red curve. Inhibition of CFTR in the continued presence of cAMP results in the blue curve. Glucagon secretion at 1mM glucose is represented by the grey area under the black curve. Activation of CFTR and cAMP amplifies glucagon secretion represented by the hatched red area under the red curve. Inhibition of CFTR results in an increased glucagon secretion (blue area under the blue curve) due to increased action potential frequency and activation of otherwise silent alpha cells. A similar pattern is observed at 6 mM glucose but the KATP conductance is so low that activation of CFTR does not silence any alpha cells.

CFTR has previously been demonstrated to be expressed in rat alpha cells^36^, and the authors speculated that impaired CFTR function would lead to increased glucagon secretion, since CFTR would normally hyperpolarize the alpha cell and hence inhibit glucagon secretion. Indeed, we could demonstrate increased glucagon secretion after inhibition of CFTR in both human and rodent islet. The alpha cell is sensitive to small changes in depolarization and the difference in membrane potential between glucose concentrations that is inhibitory (6 mM) and stimulatory (1 mM) on glucagon secretion is only ~10 mV^3^. A more negative membrane potential from which the action potentials are generated is associated with increased action potential amplitude (more voltage-dependent Na^+^ and Ca^2+^ channels are opened) until a level of hyperpolarization where action potential firing stops^3^. The bell-shaped pattern of how glucose, through the regulation of the K_ATP_ channel conductance, alternate between inhibition and stimulation of electrical activity and glucagon secretion has been demonstrated by using decreasing concentrations of diazoxid^3, 4^. Here we used the recent alpha cell model of Watts and Sherman^7^ to investigate the impact of a CFTR-dependent current on electrical activity (Fig. 7), and in Fig. 8 we put forward a suggestion on how to interpret our data based on these simulations. The black curve shows the typical bell-shaped relationship between the K_ATP_ conductance and glucagon secretion^37^, and in the graph illustrative ranges of K_ATP_ conductance representing 6 mM and 1 mM glucose are indicated. Addition of forskolin amplifies glucagon secretion due to effects on e.g. granule mobilization and priming^33, 38^, but increasing cAMP also activates CFTR. According to the model simulations of electrical activity, activation of CFTR changes the threshold for electrical activity (leftshift) and lowers it slightly due to a reduction in action potential frequency (Fig. 7). The summed effect of cAMP on priming and activation of CFTR will result in the red curve. Secretion at 1 mM glucose is thus represented by the grey area under the black line. Addition of forskolin at this glucose concentration increases secretion by moving the curve up, though some cells with K_ATP_ conductance in the upper end of the interval become inactive because CFTR is now operating, and the cells that remains active will slow down action potential firing. The net effect will result in glucagon secretion as indicated by the hatched red area. Inhibition of CFTR will reactivate cells inactivated by CFTR, accelerating action potential firing again, and secretion, which is still amplified by the cAMP effects on priming and secretion, is as indicated by the blue area. At 6 mM glucose, a similar scenario is occurring with the difference that it is only the action potential frequency that matters, since alpha cells now have a K_ATP_ conductance so low that activation of CFTR will not inactivate any of the cells. Thus, our model simulations support the hypothesis that CFTR is involved in controlling the membrane potential in pancreatic alpha cell, keeping the glucagon secretion at a balanced low level. This electrophysiological explanation applies to and corresponds to our human secretion data, while other mechanisms contribute to CFTR function in mouse alpha cells as discussed below. This interpretation would agree with CFRD patients having reduced ability to suppress glucagon secretion in combination with insufficient insulin secretion during an oral glucose tolerance test^39, 40^.

In beta cells, we have previously shown that CFTR functions as a regulator of ANO1 as a part of the machinery controlling priming of granules prior to exocytosis and release of insulin^31^. Our current data indicates that CFTR in mouse alpha cells can have this function in addition to its role as an ion channel controlling the membrane potential. In human islets, however, the main effects on glucagon secretion of CFTR-inhibition is through regulation of the membrane potential, since neither CFTR-inhibitors nor blockers of ANO1 had any effects on depolarization-induced secretion. Hence, our current and earlier^31^ data suggest that the function of CFTR in alpha- and beta-cells differs. CFTR acts mainly as an ion-channel in alpha cells influencing the membrane potential, and as a regulator in beta cells controlling exocytosis through ANO1.

In addition to the findings in alpha cells, we show that CFTR is not present in human delta cells. How can we then explain the measured effects by the CFTR inhibitors on somatostatin secretion? At low glucose, the effects of the inhibitors are absent (rodent; Fig. 3C; human; Fig. 2D). Glucagon secretion have been suggested to stimulate somatostatin secretion, thus, the small increase in somatostatin secretion at intermediate glucose in human islets (Fig. 2E) might be due to the increased glucagon secretion in presence of the inhibitors in combination with reduced paracrine effects from neighbouring beta cells (ref. 31 and Fig. 1A). In mice, at high glucose (Fig. 3D) and during depolarization-induced stimulation of somatostatin secretion (Fig. 6), the reduced response in presence of CFTR inhibitors are most likely due to paracrine effects from beta cells. Recently the peptide, Urocortin 3 (Ucn3) was shown to be co-released with insulin and to stimulate somatostatin secretion^20^. CFTR-inhibition in beta cells reduce exocytosis^31^ and theoretically this should also lead to the reduced release of Unc3, thus resulting in less somatostatin secretion. This scenario is clear in the rodent islet, but in human islets somatostatin secretion was not at all affected by CFTR inhibition. If this is due to a combination of glucagon secretion being stimulated and beta cell exocytosis inhibited or if the paracrine regulation differs between the species needs to be further investigated. It might be anticipated that the difference in islet architecture^41^ will impact the paracrine regulation.

The enhanced glucagon secretion after CFTR-inhibition might also be due to paracrine effects from neighbouring beta cells, since insulin inhibits glucagon secretion^1^. However, at glucose concentrations below 3 mM it is well established that glucagon secretion is stimulated whereas insulin secretion is inhibited. Moreover, the CFTR inhibitors had no effect on insulin release at low concentrations of glucose (ref. 31 and Fig. 1A,E). Thus, it is unlikely that the increase in glucagon release in the presence of CFTR inhibitors at low glucose concentrations is explained by paracrine insulin signals from beta cells. At high glucose, there is a possibility that the increased glucagon secretion after CFTR inhibition is partly due to reduced inhibition by insulin. However, our ion channel current measurements together with immunostainings indicate that CFTR has a higher expression in alpha cells than in beta cells. Hence, the major contribution to the enhanced glucagon secretion after CFTR-inhibition is most likely through intrinsic CFTR within the alpha cells rather than through paracrine signalling from neighbouring beta cells.

It is well established that the insulin response to an oral glucose tolerance test is delayed and reduced in CF regardless of normal or impaired glucose tolerance. Glucagon secretion is less well studied, but impaired glucagon secretion in CF has been reported^42, 43^. With decreasing glucose tolerance the ability to suppress glucagon secretion is increasingly impaired^39, 40^. Moreover, glucagon stimulates, while insulin inhibits, hepatic glucose production. CF patients have, regardless of the degree of glucose intolerance, increased hepatic glucose production^44–46^. We speculate that glucagon hypersecretion could contribute to and worsen glucose control in CF and CFRD. Our results might also have implications for type 2 diabetic patients. Studies of whether inhibition of CFTR channel activity through impaired regulation might be one of the contributing factors to the observed dysregulated glucagon secretion and failure to suppress glucagon when blood glucose increases also in these patients are warranted.

In summary, our data demonstrates the presence of functional CFTR channels in pancreatic alpha cells, and suggests CFTR to be involved in the control of the alpha cell membrane potential (ultimately) regulating glucagon secretion. Our data agree with the earlier suggestion that CFRD is associated with impaired suppression of glucagon secretion during a glucose load^39, 40^, and suggest that loss-of-function mutations of *CFTR* would impair glucagon secretion mainly through an intrinsic alpha cell mechanism.

## Methods

### Ethical statement

The use of isolated human islets from deceased donors was approved by the ethics committees in Malmö and Uppsala, Sweden. All Animal procedures were approved by the local ethical committee for use of laboratory animals in Malmö, Sweden. All experiments were performed in accordance with relevant guidelines and regulations.

### Islet isolation and cell culture

Islets from 20 human donors (F/M 9/11, age 60.3 ± 2.2, BMI 25.2 ± 0.9 kg/m2, HbA1c 5.7 ± 0.09, days in culture 4.2 ± 0.5) were provided by the Nordic Network for Clinical Islet Transplantation (Uppsala, Sweden). Human islets were hand-picked to ensure high purity. Female NMRI mice (Bolmholtgaard, Denmark) were sacrificed by cervical dislocation and islets isolated by Collagenase P (Boehringer Mannheim, Sweden) digestion as previously described^31^. For cell culture, islets were transferred to calcium-free isolation solution and dispersed into single cells and cultured in RPMI-1640 medium (SVA, Sweden) supplemented with 5 mM glucose (human) 10 mM glucose (mouse), 5% (vol/vol) fetal calf serum, 100 μg/ml streptomycin, 100 IU/ml penicillin and 2 mM L-Glutamine (Sigma-Aldrich).

### Immunohisto- and cytochemistry

#### Staining of pancreatic section

The following primary antibodies were used: mouse anti-CFTR, code: MATG-1061, dilution 1:50 (RD-Biotech, France), rabbit anti-somatostatin, code: N-SOM, dilution 1:800 (InkStar, Stillwater, MN), guinea pig anti-glucagon, code: M8707, dilution 1:2500 (Euro-Diagnostica, Sweden). Antibodies were diluted in PBS containing 0.25% BSA and 0.25% Triton X-100. Human pancreatic sections (5 μm) were incubated with primary antibodies overnight at 4 °C, followed by rinsing in PBS with 0.25% Triton X-100 for 2 × 10 minutes. Thereafter, secondary antibodies (Donkey anti-rat Texas Red, 1:400 for CFTR, donkey anti-rabbit Cy2 1:400 for somatostatin and donkey anti-guinea pig AMCA, 1:100 for glucagon) were applied for one hour at room temperature. Sections were again rinsed in PBS with 0.25% Triton X-100 and mounted in PBS:glycerol (1:1).

#### Staining of single alpha cells

Human or mouse single cells were seeded on poly-L-lysin (Sigma Aldrich, Sweden) coated dishes and cultured overnight. Cells were fixed and stained as described elsewhere^31^. Mouse monoclonal anti-CFTR (MATG-1061, RD-Biotech, France) and Guinea pig polyclonal anti-glucagon (EuroProxima, Netherlands) were used, and secondary antibodies specific for mouse conjugated to Cy3 and guinea pig conjugated to Cy5 (Jackson, UK). Unspecific binding of the secondary antibodies was excluded by parallel experiments in the absence of the primary antibodies. Immunofluorescence was detected with a confocal microscopy (META 510, Zeiss, Germany) using the 525 nm and 633 nm lines for excitation. Mean intensity analysis was performed using Zen software (version 2009, Zeiss, Germany). The same laser setting was retained between experiments enabling comparison of experiments performed at different occasions. Localization of CFTR was analyzed as described elsewhere^31^. In short, the ratio between the mean fluorescent intensity in the plasma membrane region (P1) and the cytosolic region (P2) was estimated. The width of the plasma membrane region was decided to 0.5 μm.

### Patch-clamp recordings

EPC10 amplifier and 8.80 pulse software (HEKA, Germany) was used to evoke and record currents and changes in membrane capacitance on single islet cells. Mouse alpha cells were distinguished from beta cells by the electrophysiological properties of the Na^+^ channel inactivation^47^. Current and capacitance measurements were performed at RT and 32–33 °C, respectively.

Single channel currents measurements were performed using the cell-attached mode. In these experiments both the pipette and bath solutions contained: 140 mM NMDG-Cl, 2 mM CaCl_2_, 1 mM MgCl_2_, 10 mM Hepes, 16.7 mM glucose and 10 mM Manitol (pH 7.4), supplemented with 10 μM forskolin and 25 μM GlyH-101 (Calbiochem, USA) as indicated. During recording the currents were filtered at 3 kHz and digitized at 10 kHz. On playback, traces were filtered at 500 Hz and analysed using Heka software.

Whole-cell currents and changes in membrane capacitance were measured using the whole-cell configuration. To inhibit K^+^-fluxes in the current measurements, the intracellular solution was free from K^+^-ions and the extracellular solution was supplemented with the K^+^-channel blocker TEACl. Extracellular solution contained: 118 mM NaCl, 20 mM TEACl, 5.6 mM KCl, 2.6 mM CaCl_2_, 1.2 mM MgCl_2_, 5 mM HEPES, 3 mM L-glucose (current measurement)/5 mM L-glucose (capacitance measurement) pH 7.4 (NaOH), supplemented with 10 μM forskolin and/or 10 μM CFTRinh-172 (both from Sigma Aldrich, USA) and/or 25 μM GlyH-101 (Calbiochem, USA), as indicated. The intracellular solution contained: 125 mM CsOH, 125 mM Glutamate, 10 mM CsCl, 10 mM NaCl, 1 mM MgCl_2_, 3 mM Mg-ATP, 5 mM HEPES (pH 7.15 with CsOH). For the current measurements 4 mM EGTA was included to the intracellular solution and for capacitance measuremenst 0.05 mM EGTA and 0.1 mM cAMP was added.

### Hormone secretion measurements

Glucagon and somatostatin secretion was measured in static batch incubations described previously^33^. Briefly, batches with 12 islets (in quadruplicates) were pre-incubated in KRB supplemented with 1 mM glucose for 30 min followed by 1 h incubation at 1, 2.8, 6 or 16.7 mM glucose and in the presence of 50 mM KCl, 10 μM forskolin, 40 μM CFTRinh-172, 20 μM or 50 μM GlyH-101 (Calbiochem, USA) and 50 μM T16Ainh-AO1 (Calbiochem, USA) as indicated. When 50 mM KCl was added to the solution equimolar NaCl was removed. Forskolin, CFTRinh-172, GlyH-101 and T16Ainh-AO1 were dissolved in DMSO to a final concentration of <0.1% DMSO in the experiment. Glucagon and somatostatin secretion was measured using radioimmunoassay kit (Millipore, MA, USA and Euro-Diagnostica, Malmö, Sweden, respectively).

### Mathematical model

We used the recent mathematical model of electrical activity in pancreatic alpha cells by Watts and Sherman^7^. This model is based on electrophysiological characterizations of mouse alpha cells located in intact islets^47, 48^, and we based our simulations on the version of the model without store-operated currents operating (Fig. 1 in﻿ ref. 7). We inserted a CFTR current into the model. The CFTR current was represented as a passive current, *I*_CFTR_ = *g*_CFTR_ (*V*-*V*_Cl_), where the conductance *g*_CFTR_ = 0.100 nS and the Cl^−^ reversal potential *V*_Cl_ = −60 mV were based on the experimental results presented here. The value for *g*_CFTR_ was slightly lower than estimated from our experiments in order to produce robust electrical activity. This we attribute to the different experimental conditions, in particular the use of isolated alpha cells for the characterization of the CFTR current, in contrast to the use of intact islets for the parameters of the other currents in the model, and in secretion experiments. All expressions and parameters are as in Watts and Sherman^7^, except the parameters for the leak current, which were set to *V*_L_ = 0 mV, and *g*_L_ = 0.150 nS or *g*_L_ = 0.200 nS for 1 or 6 mM glucose, respectively. Simulations were performed using the code solver in the XPPAUT software. Computer code can be found in the supplementary material.

### Statistical analysis

Data are presented as mean ± SEM of N number of individuals or independent experiments or n number of cells. In the hormone secretion experiments, N number of human donors or experiments (islets from 3–4 mice were pooled for each secretion experiment) was used for the statistical analysis using 2-way ANOVA. All patch-clamp measurements and confocal immunostainings were analysed as n number of cells using Students t-test. p < 0.05 was considered statistically significant.

## Electronic supplementary material

Supplementary Figure 1

Supplementary Information
