# Supplementary material for: CFTR is involved in the regulation of glucagon secretion in human and rodent alpha cells
Source: Sci Rep. 2017 Mar 7;7:90. doi: 10.1038/s41598-017-00098-8 (PMC5428348; doi:10.1038/s41598-017-00098-8)
Supplement: Supplementary file 1 — Supplementary Figure 1 [file 41598_2017_98_MOESM1_ESM.pdf]

Supplementary material

CFTR is involved in the regulation of glucagon secretion in human and rodent alpha cells

Anna Edlund[1*], Morten Gram Pedersen[2], Andreas Lindqvist[3], Nils Wierup[3], Malin Flodström-Tullberg[4], Lena Eliasson[1*]

[1]Unit of Islet Cell Exocytosis, Lund University Diabetes Centre, Department of Clinical Sciences in Malmö, Lund University, CRC, SUS Malmö, Malmö, Sweden

[2]Department of Information Engineering, University of Padova, Via Gradenigo 6/B, 35131, Padova, Italy

[3]Unit of Neuroendocrine Cell Biology, Lund University Diabetes Centre, Department of Clinical Sciences in Malmö, Lund University, CRC, SUS Malmö, Malmö, Sweden

[4]Center for Infectious Medicine, Department of Medicine, Karolinska Institutet, Huddinge University Hospital, Stockholm, Sweden

*Address correspondence and requests for reprints to: Anna Edlund and Lena Eliasson, Unit of Islet cell Exocytosis, Lund University Diabetes Centre, Department of Clinical Sciences in Malmö, Lund University, Jan Waldenströms gata 35, 205 02 Malmö, Sweden. Phone +46 40 391153. Fax: +46 40 391222. E-mail: anna.edlund@med.lu.se and lena.eliasson@med.lu.se

Supplementary Figure 1

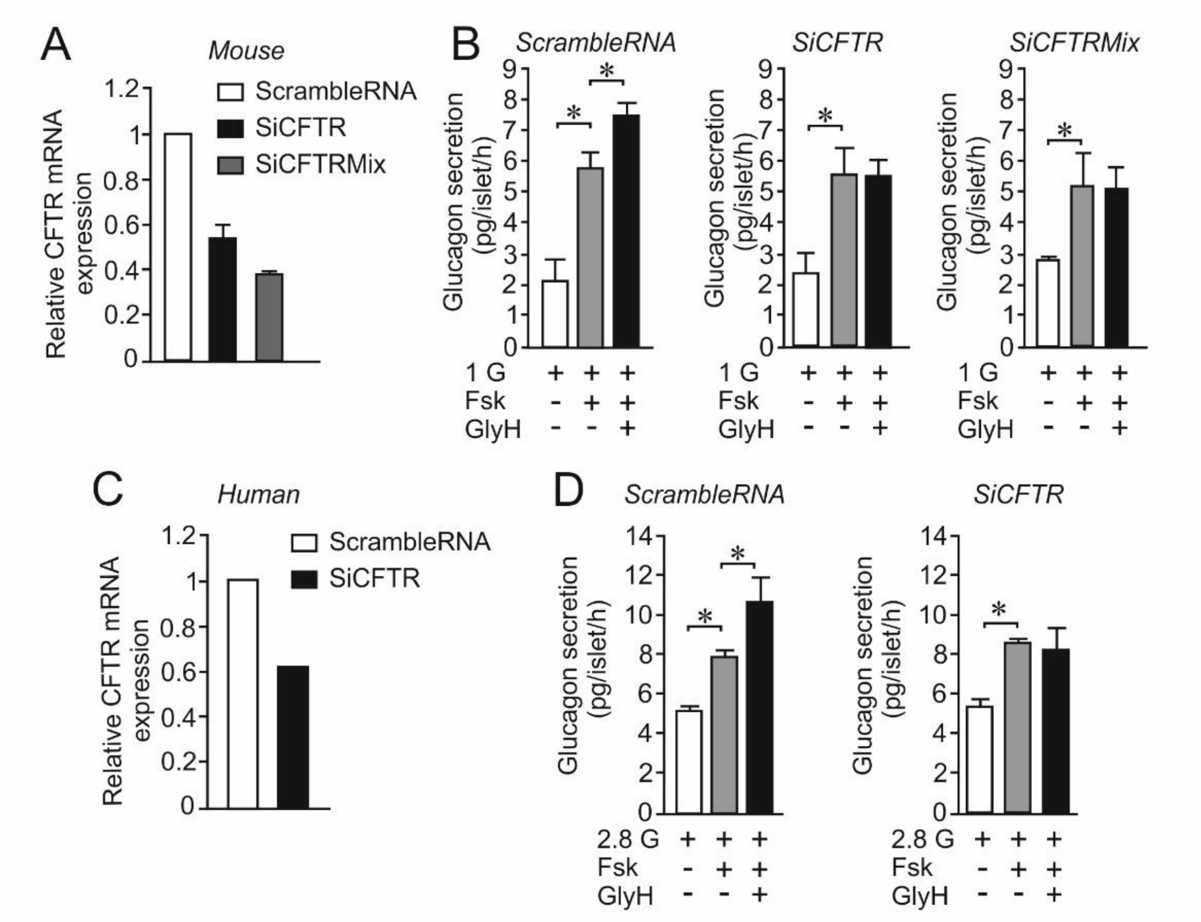

Supplementary figure 1. Knock-down of CFTR using SiRNA transfection of intact mouse and human islets demonstrate that effects on glucagon secretion by GlyH-101 act through CFTR. (A) In mouse islets using one single SiRNA targeting CFTR (SiCFTR) caused a 46±6% knock-down of CFTR mRNA (black bar, N=4) and a mixture of three different siRNA (SiCFTRMix) decreased CFTR mRNA expression by 62±0.6% (grey bar, n=3) compared to scramble RNA (ScrambleRNA; white bar, n=4). **(B)** Glucagon secretion measured on islets transfected with scramble RNA (left; N=4 biological replicates with 8-9 technical replicates in each), one single CFTR siRNA (middle; N=4 biological replicates with 8-9 technical replicates in each) and three different CFTR siRNA. (siCFTRMix; right; n=3 biological replicates with 8-9 technical replicates in each). Islets were incubated for 1h at 1 mM glucose (1G) in the presence or absence of 10 µM forskolin (FSK) and 50 µM GlyH-101 (GlyH). Data shows that GlyH is unable to stimulate glucagon secretion in siCFTR/siCFTRMix cells. Thus, GlyH effects glucagon secretion mainly through inhibition of CFTR. (C) Knock-down of CFTR in islets from one human donor. Transfection of siRNA targeting CFTR caused a 40% knock-down of CFTR mRNA. (D) Glucagon secretion from human islets transfected with scramble RNA (left; n=4 technical replicates) or CFTR siRNA (right; n=4 technical replicates). Islets were incubated for 1h at 2.8 mM glucose (2.8 G) in the presence or absence of 10 µM forskolin (FSK) and 50 µM GlyH-101 (GlyH). Data are presented as mean±SEM, *p<0.05.

Supplementary Material and methods

Transfection of islets with SiRNA

Human islets were hand-picked directly into RPMI-1640 cell culture media (5 mM glucose) and mouse islets were hand-picked in HANKS balanced salt and cultured over-night in RPMI-1640 cell culture media (10 mM glucose) supplemented with 0.25 µg/ml Fungizone (Gibco™). The

islets were transfected using Lipofectamine RNAiMAX (Invitrogen, CA, USA), opti-MEM (Gibco^{TM}) and either 50 nM scramble RNA (silencer select negative control 2, Ambion, USA), Hu SiCFTR (s2945, Ambion, USA), Mu SiCFTR (s63911, s63912, s63910, Ambion, USA) according to manufactures protocol. After 48h the islets were re-transfected without Fungizone in the cell culture media. After 96h incubation islets were assayed for glucagon secretion and mRNA expression. CFTR mRNA expression was quantified by rtq-PCR (Quantstudio^{TM} 7 Flexsystem) using Hs00357011 (human) and Mm 00445197 (mouse). CFTR mRNA expression were normalized to HPRT1 (HPRT1(DQ) OligoMix #4332657, Mm00446968) and PPIa (Human PPIA(DQ) OligoMix #433337637) or PPIb (Mouse Mm00478295) (All from Applied Biosystems, UK.). The use of isolated human islets from deceased donors was approved by the ethics committees in Malmö and Uppsala, Sweden and performed in accordance with relevant guidelines and regulations.
